# Human oocytes image classification method based on deep neural networks

**DOI:** 10.1186/s12938-023-01153-4

**Published:** 2023-09-21

**Authors:** Anna Targosz, Dariusz Myszor, Grzegorz Mrugacz

**Affiliations:** 1 Department of Histology and Embryology, Faculty of Medical Sciences, Medical University of Silesia, 18 Medyków St, 40-752 Katowice, Poland; 2 Center for Reproductive Medicine Bocian, 26 Akademicka St, 15-267 Białystok, Poland; 3 Institute of Computer Sciences, Silesian University of Technology, 16 Akademicka St, 44-100 Gliwice, Poland

**Keywords:** IVF, Human oocyte, Classification, Artificial intelligence, Machine learning, Deep neural network

## Abstract

**Background:**

The effectiveness of in vitro fertilization depends on the assessment and selection of oocytes and embryos with the highest developmental potential. One of the tasks in the ICSI (intracytoplasmic sperm injection) procedure is the classification of oocytes according to the stages of their meiotic maturity. Oocytes classification traditionally is done manually during their observation under the light microscope. The paper is part of the bigger task, the development of the system for optimal oocyte and embryos selection. In the hereby work, we present the method for the automatic classification of oocytes based on their images, that employs DNN algorithms.

**Results:**

For the purpose of oocyte class determination, two structures based on deep neural networks were applied. DeepLabV3Plus was responsible for the analysis of oocyte images in order to extract specific regions of oocyte images. Then extracted components were transferred to the network, inspired by the SqueezeNet architecture, for the purpose of oocyte type classification. The structure of this network was refined by a genetic algorithm in order to improve generalization abilities as well as reduce the network’s FLOPs thus minimizing inference time. As a result, $\overline{Acc}$ at the level of 0.964 was obtained at the level of the validation set and 0.957 at the level of the test set. Generated neural networks as well as code that allows running the processing pipe were made publicly available.

**Conclusions:**

In this paper, the complete pipeline was proposed that is able to automatically classify human oocytes into three classes MI, MII, and PI based on the oocytes’ microscopic image.

## Background

Quality and development potential of oocytes are one of the most important factors determining the success of assisted reproductive technology (ART) [1, 2]. For the ICSI (intracytoplasmic sperm injection) procedure, even several dozen oocytes at various stages of their meiotic maturity are obtained. Approximately, 80$\%$ of the collected oocytes are at the stage of metaphase II meiotic division (MII), remaining oocytes are at the stage of metaphase I (MI), prophase I meiotic division (PI), degenerated cells and abnormal cells [3, 4]. The degree of oocyte maturity is determined on the basis of the presence of the first polar body (FPB) and germinal vesicle (GV) structures [3, 5]. Despite numerous guidelines, e.g., ESHRE, regarding the unification of the assessment of oocytes and embryos, it has still not been possible to achieve unanimity in their assessment. Proper assessment of oocyte is important in terms of successful fertilization, embryo development and achieving pregnancy. A research for new methods to simplify the selection of oocytes with the highest development potential is in progress [6].

**Fig. 1:**
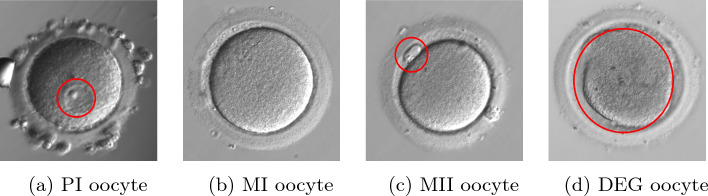
Example of human oocyte images in the stage of prophase I (PI) metaphase I meiotic division (MI), metaphase II (MII), and degenerated cells (DEG)

Figure 1 presents particular classes of oocyte maturity. PI oocyte (Fig. 1a)—oocytes at this stage are not used in the ART due to their meiotic immaturity. The microscopic image allows to differentiate zona pellucida (ZP) surrounding the perivitelline space (PVS) and cytoplasm with a clearly marked GV. The presence of GV is typical only for this particular stage of meiotic development. The microscopic image of MI oocyte (Fig. 1b) show begins for an oocyte initially in prophase I with the nuclear envelope breakdown (NEBD). Image of MI oocyte allows to differentiate ZP surrounding the PVS and cytoplasm. Oocytes at this stage are also not used in the ART procedure due to their meiotic immaturity. MII oocyte (Fig. 1c) is oocyte in metaphase II of the second meiotic division. Oocytes at this stage are mature oocytes. On the microscopic image, ZP surrounding the cytoplasm and PVS with isolated first polar body FPB can be distinguished. The appearance of FPB is typical only for this stage. These oocytes can be intended for fertilization. Sometimes degenerated oocytes (Fig. 1d) can be observed. Oocyte degeneration can occur at any stage of meiotic development, therefore it can contain different characteristic areas occurring in different stages. The cytoplasm in the microscopic image changes its granularity; it is clearly darker and may lose oolemma continuity and oval shape.

In some countries, the number of oocytes subjected to the procedure is limited. This approach is related to prohibited cryopreservation of embryos on ethical grounds [7–9]. One of the major problems at an IVF laboratory is choosing adequate-quality oocytes. That is the reason for seeking non-invasive methods for assessing the quality of oocytes and embryos [10]. In our previous work, we presented the method of semantic segmentation of oocytes, taking into account the identification of 13 morphological structures. Very good results of the weighted intersection over union (IoU) 0.897 and global accuracy 0.93 for test patterns were obtained [11]. In the hereby work, we present the next step of the method for the automatic classification of oocytes depending on the obtained meiotic maturity PI, MI, MII. Oocyte quality assessment is based mainly on extracellular and intracellular morphological features. Although the oocyte’s development potential is identified as the most important factor in determining the success of ART, the assessment itself still remains subjective. Many attempts have been made to define the prognostic factors based on oocyte morphology. [12–17]. The application of artificial intelligence (AI) in medicine brings new possibilities for the selection of oocytes. Manna et al. used the neural network to analyze the texture of oocyte images in relation to the quality of the obtained embryo. As a result of the procedure, an accuracy equal to 0.80 was obtained [18]. The meta-analysis by Setti et al. indicated lowered results of the ICSI procedure for oocytes with large FPB, presence of refractile bodies, vacuoles and large PVS [19]. Convolutional neural networks (CNN) was applied by Kanakasabapathy et al. to predict fertilization potential from oocyte images [20]. CNN deep learning was also used by Dickinson et al. to localize the FPB and determine the sperm injection location during the ICSI procedure with an accuracy of 98.9 $\%$ [20].

## Results

For the purpose of oocyte class determination, two structures based on deep neural networks were applied. DeepLabV3Plus [21] was responsible for the analysis of oocyte microscopic images in order to extract specific regions of oocyte images. Then extracted components were transferred to the network inspired by the SqueezeNet architecture [22] for the purpose of oocyte type classification. The structure of this network was refined by a genetic algorithm in order to improve generalization abilities as well as minimize the number of learnable parameters which could potentially lead to the reduction of the network’s FLOPs and inference time.

### Oocytes classification network refinement

Searching for the best network structure had three phases: (1) initial evaluation of the number of layers; (2) selection of input features; and (3) final refinement of network structure.

#### Classification network structure refinement

The DNN structure for oocyte classification applied in this research was inspired by the SqueezeNet concept.

SqueezeNet contains convolutional layers that are followed by ReLu activation functions. For the reduction of feature map resolution max-pooling layers are applied. Key components of the SqueezeNet structure are repetitive inception blocks, each containing two patches one with 1 × 1 2D convolution filters, and the other with 3 × 3 2D convolution filters. Then dropout layer is applied in order to improve network generalization abilities, it is followed by 1 × 1 2D convolution filters and the global average pooling layer. The output of the network is processed by the Softmax function.

Initially, the SqueezeNet structure, implemented for the purpose of oocyte type determination, contained 4 inception blocks. The structure was trained with preprocessed labeled data (see section "Methods"). All available input features were utilized for the purpose of initial network structure determination. The best result of the mean accuracy coefficient $\overline{Acc}$ obtained on the validation set was equal to 0.65. Analysis of the loss function plot, for the training and validation sets, revealed the occurrence of an overfitting phenomenon. The values of the loss function obtained on the training set were significantly lower than the values of the loss function obtained on the validation set. Therefore, a reduction of the number of inception layers was done. Results are presented in Table 1. The benefits of network structure simplification are clearly visible. Based on obtained results, the conclusion can be made that one or two inception layers should be employed. In addition, with the reduction of the number of inception layers, the number of DNN’s FLOPs is reduced.

**Table 1:** Performance of classification network structures with various number of inception layers, 3-time SRRS was applied

Number of inception layers	Accuracy ($\overline{Acc}$)	FLOPs
4	0.65	3578.26e6
3	0.7	2530.53e6
2	0.99	1788.40e6
1	0.99	1526.46e6
0	0.94	1028.52e6

#### Classification network input fine-tuning

There are components of the oocyte that do not influence its type determination (e.g., cumulus/corona cells); in addition, the presentation of superfluous information on the network input can be detrimental to the quality of network classification.

In order to find the optimal DNN input composition, a set of experiments was conducted. During the first phase, the goal was to determine oocyte structures that have the greatest impact on network accuracy in the task of MI, MII, and PI discrimination. Therefore, for each individual oocyte feature as an input, a separate set of DNNs was trained. The structure selected in the previous section was employed. For a single input layer selected structure exhibits a calculated FLOPs count of 879.23e6. The obtained results are presented in Table 2. Features for which $\overline{Acc} > 0.55$ were selected. Two structures fulfill this condition: the FPB and the GV. When the input dataset contains only these two features mean accuracy equal to 0.92 was obtained. In the next phase remaining layers were added individually to the dataset, and for each combination of FPB/GV + one remaining feature ANN was trained. Results are presented in Table 3. When the fragmented first polar body (FFPB) was added to the set $\overline{Acc}$ on the validation set at the level of 0.99 was obtained.

The FFPB was introduced at the level of manual segmentation, in order to introduce specific information about FPB region characteristics,

FFPB and the FPB region can be treated as a single region. Therefore, in further training, FPB and FFPB regions were merged and constituted a single input feature referred to as FPB. Such DNN structure exhibits a calculated FLOPs count of 879.23e6.

**Table 2:** Mean accuracy coefficient $\overline{Acc}$

ID	Input layer	Accuracy
1	Oocyte image	0.41
2	Clear cytoplasm	0.49
3	Diffuse cytoplasmic granularity	0.40
4	Cytoplasmic granular area	0.47
5	Smooth endoplasmic reticulum cluster	0.33
6	Vacuoles	0.36
7	Dark cytoplasm	0.35
8	First polar body	0.58
9	Multi-polar body	0.33
10	Fragmented first polar body	0.39
11	Second polar body	0.33
12	Perivitelline space	0.50
13	Zona pellucida	0.54
14	Germinal vesicle	0.66
15	Cumulus /corona cells	0.33

Network input: class specified in the table. Output: oocyte type (PI/MI/MII)

**Table 3:** Mean accuracy coefficient $\overline{Acc}$

ID	Input layer	Accuracy
1	Oocyte image	0.93
2	Clear cytoplasm	0.93
3	Diffuse cytoplasmic granularity	0.90
4	Cytoplasmic granular area	0.93
5	Smooth endoplasmic reticulum cluster	0.94
6	Vacuoles	0.92
7	Dark cytoplasm	0.93
9	Multi-polar body	0.92
10	Fragmented first polar body	0.99
11	Second polar body	0.93
12	Perivitelline space	0.95
13	Zona pellucida	0.91

Network input: first polar body, germinal vesicle + class specified in the table. Output: oocyte type (PI/MI/MII)

#### Classification network structure fine-tuning

Neural architecture fine-tuning based on a genetic algorithm was applied in order to minimize the number of network learnable parameters and improve network generalization abilities. The structure of the network and input features, determined in previous sections, were utilized. The chromosome, defining DNN structure, consisted of positive integer values that represented the number of filters at respective 2D convolution layers (there are $L_n=6$ such layers in the structure). The minimum number of 2D convolution filters in the DNN layer was set to 2 (FLOPs count 0.15e3), and the maximum number of 2D convolution filters in the DNN layer was set to 128 (FLOPs count 4340.30e6), in order to speed up the refinement process only even values were allowed.

The objective function OF of the genetic algorithm consisted of two parts. It accommodated the oocyte type classification quality coefficient ($\overline{Acc}$) as well as the number of learnable parameters possessed by the network (i.e., the number of 2D convolution filters). The function was defined as follows:

1 $$\begin{aligned} \text{OF}{} = 1 - \overline{Acc} + \frac{\sum (F_k)}{F_{max}}, \end{aligned}$$

where $F_k$—number of filters in *k* 2D convolution layer, $F_{max}$ is the maximum number of 2D convolution filters that can be exploited within the structure (it was equal to 128*6). The genetic algorithm was running for 10 epochs, each epoch contained 10 individuals, elite selection strategy with a single best individual promoted to the next epoch was utilized.

As a result, a minimal DNN structure was generated that was able to obtain the accuracy of classification ($\overline{Acc}$) equal to 1 on the validation as well as on the test dataset. At the same time, such structure exhibits a calculated FLOPs count of 160.60e6; thus, there is a reduction of FLOPs in the relation to the best network structure presented in Table 3. In addition, the structure was also able to correctly assign every element of the combined train, validation and test set to MI/MII/PI class. Detailed information about the internals of the selected structure is provided in Table 4. The quality of the network was confirmed with 10-time SRRS. The best version of the generated structure during each repetition of SRRS was saved for further validation.

**Table 4:** Network structure

Name	Type	Activations	Parameters
inputLayers	Input	590 × 590 × 2	
conv1	Convolution	294 × 294 × 32	Conv 2D 3 × 3 stride [2 2] padding [0 0 0 ]
relu_conv1	ReLu	294 × 294 × 32	
conv2	Convolution	146 × 146 × 16	Conv 2D 1 × 1 stride [1 1] padding [0 0 0]
relu_conv2	ReLu	146 × 146 × 16	
pool1_conv2	Max pooling	72 × 72 × 16	3 × 3 stride [2 2] padding [0 0 0]
conv4	Convolution	72 × 72 × 8	Conv 2D 1 × 1 stride [1 1] padding [0 0 0]
relu_conv4	ReLu	72 × 72 × 8	
res_conv5_1	Convolution	72 × 72 × 28	Conv 2D 3 × 3 stride [1 1] padding [0 0 0]
relu_conv5_1	ReLu	72 × 72 × 28	
res_conv5_2	Convolution	72 × 72 × 28	Conv 2D 1 × 1 stride [1 1] padding [0 0 0]
relu_conv5_2	ReLu	72 × 72 × 28	
depth_concat1	Depth concatenation	146 × 146 × 56	
pool_depth_concat1	Max pooling	36 × 36 × 56	3 × 3 stride [2 2] padding [0 1 0 1]
conv6	Convolution	36 × 36 × 4	Conv 2D 1 × 1 stride [1 1] padding [0 0 0]
relu_conv6	ReLu	36 × 36 × 4	
conv7	Convolution	36 × 36 × 4	1000 1 × 1 × 48 stride [1 1] padding [0 0 0]
relu_conv7	ReLu	36 × 36 × 4	
global_pool	2D global avg pooling	1 × 1 × 4	
fullyConnected1	Fully connected	3 outputs	
softMax_output	Softmax	3	

### Oocyte semantic segmentation network

In order to obtain a fully automated pipeline for oocyte classification, a structure that can extract information about GV and FPB/FFPB regions was required. The decision was made to utilize DeepLabV3Plus because it is currently a state-of-the-art model in the area of semantic segmentation.

The utilized model employed resnext101-32x8d encoder that was pre-trained on imagenet dataset [23].

The input of the segmentation DNN accepted 512x512px RGB images. During the training process, augmentation techniques were applied, and images constituting the training set were randomly rotated, in addition, random brightness modification was applied with PyTorch ColorJitter method (brightness = 0.4). The training process run for 200 epochs.

For the purpose of the best version of segmentation DNN selection, a quality measurement based on the combination of the DNN ability to detect GV/FPB regions and the *IoU* coefficient was applied. An assumption was made, that region was detected if at least 100 pixels of the region were marked correctly by the segmentation network. Then, for instances of DNN with the highest rate of detected regions (obtained during a single run of SRRS), *IoU* was applied in order to select the best network variant. Results of 3-time SRRS point out that all trained models had instances that were able to successfully detect almost all regions of oocytes from the validation set (detection ratio DR at the level of 0.93). The $\overline{IoU}$at the level of the validation set was equal to 0.75 and the $\overline{IoU}$at the level of the test set was equal to 0.66. The instance of segmentation network versions with the highest DR (on the appropriate validation set) and *IoU*, respectively, was selected, in order to generate masks for the purpose of classification network evaluation.

### Oocyte classification performance evaluation

Classification networks trained in previous sections were tested on data generated by the best version of the segmentation network. In order to confirm the validity of the approach three sets were analyzed (a) original validation sets (b) original test sets (c) all oocytes. In the scenario with original validation and test sets, masks for validation, as well as test, sets specific for a given SRRS run, employed during classification network training, were created by trained DeepLabV3Plus structure. So each classification network variant was evaluated on the original list of oocytes from the validation and test set, for which masks were generated by the segmentation network structure. As a result $\overline{Acc}$ at the level of 0.964 was obtained at the level of validation and 0.957 at the level of the test set. In addition, masks for all oocytes were created by DeepLabV3Plus. Classification networks obtained $\overline{Acc}$ equal to 0.98 at the level of this set (see Table 5).

**Table 5:** Quality coefficient of classification DNN obtained on the manually segmented dataset and on dataset segmented by DeepLabV3Plus

	Manually labeled dataset			DNN labeled dataset		
	Validation	Test	All	Validation	Test	All
$\overline{Acc}$	1	1	1	0.96471	0.95699	0.98253
$\overline{TPR}$	1	1	1	0.92692	0.88291	0.91532
$\overline{PPV}$	1	1	1	0.91587	0.86816	0.95112
$\overline{FDR}$	0	0	0	0.084126	0.13184	0.048878
$\overline{f1}$	1	1	1	0.91364	0.871	0.93246

## Discussion

The research was carried out to fine-tune the set of input parameters of the classification network. On the one hand, it was decided to check which morphological areas of cells affect the determination of the class of meiotic maturity of oocytes, additionally, it was possible to reduce the computational complexity. It has been experimentally verified that the presence of FPB and GV areas in oocyte images is sufficient for oocyte type determination, which is consistent with biological knowledge. It can be concluded that the remaining areas to determine the maturity class are not necessary, but the analysis of these results will be used in the future to determine the developmental potential of MII oocytes.

A qualitative analysis of segmentation errors was performed. Seven of the incorrectly segmented oocytes images were assessed by a specialist as MI class cells (without FPB area), whereas the automatic classifier could mark them as MII cells because FPB areas were found. Four of the images could be classified as PI instead of MI due to the fact that GV areas exist on the predicted masks. The segmentation network did not find FPB area on one image and GV on another one. Table 6 presents examples of incorrectly segmented images, along with their masks of FPB or GV marked by an expert and segmentation network.

Incorrect segmentation in all groups is caused by poor image quality or insufficient denudation. In addition, incorrect segmentation in group I is caused by the incorrect determination of granularity in PVS. The error in group II is caused by the poor quality of the photo with visible grains on the cell surface. Group III error is also caused by the bad focus of the photo. The misclassification of group IV is related to the indistinct edge of the GV structure. The analysis of these errors indicates even greater accuracy of the algorithm, but at the same time underlines the sensitivity of this method to a photo quality.

**Table 6:** Oocytes images wrongly classified by DNN system

Group I: expert-MI, prediction-MII	

Group II: expert-MI, prediction-PI	

Group III: expert-MII, prediction-MI	

Group IV: expert-PI, prediction-MI	

## Conclusion

In this paper, the complete pipeline was proposed that is able to automatically assign human oocytes into one of three classes MI, MII, and PI based on the oocytes’ microscopic image. The proposed pipeline was able to obtain high accuracy of classification (Table 5). Obtained results were confirmed with dataset repeated random subsampling techniques. The pipeline is composed of two artificial deep networks, the first one is responsible for the extraction of features based on oocyte image. The second invokes tasking the obtained features and assigning the oocyte into proper classes. The first network was based on DeepLabV3Plus architecture: in this research, it was applied to extract the information concerning the location of GV and FPB components. Output generated by this network was processed by a classification network that was inspired by SqueezeNet architecture and was further improved by genetic algorithms. The provided analysis points out a few interesting conclusions. For the analyzed dataset of 766 oocytes, GV and FPB components were both able to assign oocytes to proper classes. Thus it was sufficient to train the segmentation network structure that was tweaked specifically for the purpose of detection of these two oocytes’ components. Such an approach facilitated the process of the training and allowed for the high-fidelity detection of these small-scale structures. GV and FPB regions, during regular DNN training, could be omitted easily by the model because these zones take only a small fraction of the whole oocyte structure (GV 11 % and FPB 12 % of total oocyte surface and 0.1 % 0.3 % of the whole image). When all oocyte components must be extracted by a single DNN, some techniques of data augmentation and loss function coefficient modifications can be applied that mitigate this effect, but they do not guarantee successful training.

It is worth mentioning that, in order to obtain proper classification, the quality of obtained material is important. If obtained microscopic images are blurry, or contain artifacts such as burnouts, oocyte can be assigned to the incorrect class. The obtained results between SRRS trials are consistent. All classification networks (selected from each SRRS run) when employed on the whole dataset, assigned incorrectly the same set of oocytes. Importantly, such results were obtained on the dataset processed by the DeepLabV3Plus structure for semantic segmentation. It confirms that the applied methodology, parameters, augmentation techniques as well as trained structure were well accommodated to the analyzed problem.

## Methods

**Fig. 2:**
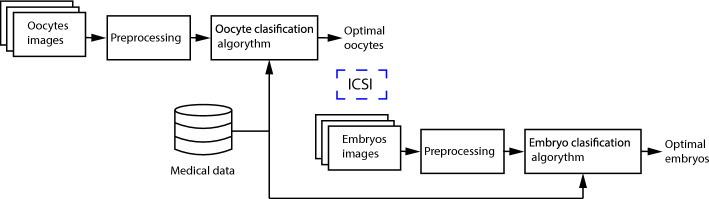
Methodology of selecting optimal oocytes and embryos [11]

The presented method is done according to the suggested methodology of selecting optimal oocytes and embryos, described in the previous article [11] and schematically presented in Figure 2. The hereby work focuses on the use of deep learning techniques for the automatic classification of oocytes’ meiotic maturity, to be successively subjected to the ICSI procedure.

**Fig. 3:**
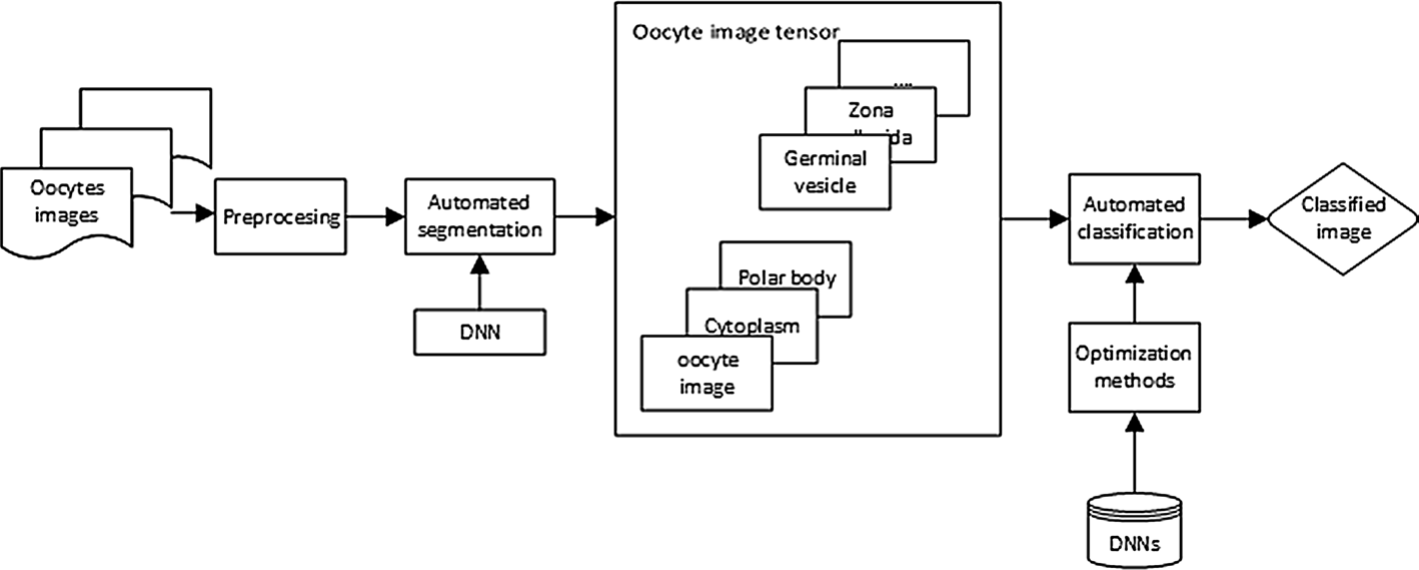
Oocyte classification method

Oocytes classification methods are presented in Fig. 3. In this method, the input is a photo of the oocyte. In the first phase, the deep neural network segments the relevant areas of the oocyte image. The selected areas are the input of the second neural network. The obtained tensor is subject to automatic classification. The structures and parameters of the neural networks used in this task were fine-tuned.

### Deep neural network models

The classification network applied in this research was inspired by SqeezeNet architecture [22]. The authors of the structure designed it in order to compete with AlexNet in the area of accuracy. The goal of the authors was to obtain a significant reduction in the number of structure parameters. The architecture was devised for image classification purposes. Authors of the network proved that the trained version was able to assign images to 1000 object categories. Matlab was employed for the purpose of network implementation and testing. The segmentation network, utilized in this work, was based on DeepLabV3Plus [21]. It is a state-of-the-art model for semantic image segmentation. It incorporates encoder–decoder architecture in which the encoder extracts high-level features and the decoder regains spatial information that was lost by the encoder. It utilizes atrous spatial pyramid pooling for the purpose of capturing features at different scales. Python library with Neural Networks for Image Segmentation based on PyTorch called Segmentation Models was employed for the purpose of the training as well as network quality evaluation [24].

The cross-entropy loss function and ADAM optimization algorithm were applied during the training process.

### Data set preparation

Oocytes have been collected from 100 patients (average age of 32 ± 10 years) subjected to ICSI procedure. In total, 766 images of oocytes have been used, including 663 images of oocytes classified as MII, 44 as MI, and 59 as PI. The patients were subjected to hormonal stimulation. The ovarian stimulation protocol was chosen based on the clinical picture. After collection, the cumulus–oocyte complex (COC) was incubated for 2−5 h in culture medium (SAGE 1-Step™, Origio CooperSurgical Companies) in an incubator at $37^{\circ }$C, $6\%$ CO$_2$. Oocytes were subjected to denudation of granular cells by exposure to 80 IU/ml hyaluronidase (GM501 Hyaluronidase; Gynemed Germany) for 1 min and mechanically cleaned. Pictures were taken with use of an inverted light-microscope (Olympus®, IX51/IX70) at x200 magnification, using a camera (Oosight CCD Camera Module) and Oosight$^\circledR$ Meta software (Hamilton Thorne, Inc.). The recorded image may contain one or more oocytes and micromanipulation needles, on the condition that it did not affect the individual shape of each oocyte. In order to prepare learning patterns, each image underwent manual segmentation. The segmentation was carried out employing the Image Labeler application available as part of MATLAB®R2019b software.

#### Classification network

Before the training phase, labeled data were preprocessed. Inverted light-microscope returns grey-scale bmp files with a resolution of 1392x1024 px. Each pixel is represented by 24 bits. For each labeled oocyte a tensor is created, the first layer contains a grey-scaler representation of the oocyte. It is followed by layers that consist of binary masks for oocyte regions. Layers were organized in the following order 1: oocyte image, 2: clear cytoplasm, 3: diffuse cytoplasmic granularity, 4: cytoplasmic granular area, 5: smooth endoplasmic reticulum cluster, 6: vacuoles, 7: dark cytoplasm, 8: first polar body, 9: multipolar body, 10: fragmented first polar body, 11: second polar body, 12: perivitelline space, 13: zona pellucida, 14: germinal vesicle, 15: Cumulus /corona cells.

Over 95 $\%$ of oocytes’ images have dimensions smaller than 550x550px. In order to normalize artificial neural network input Oocytes that had representation greater than 550x550px, were downscaled with their respective masks to the resolution 550x550 using nearest-neighbor interpolation. As a result, a tensor with the dimensions 550x550x14 is obtained. Further, the number of layers was limited during the network structure fine-tuning process.

The original dataset consists of 44 MI oocytes 663 MII oocytes and 59 PI oocytes. The dataset is imbalanced, there is a significant overrepresentation of MII oocyte types at the same time MI and PI are rare. Direct utilization of such a set during the artificial neural network training process can lead to incorrect predictions at the level of underrepresented classes.

For the classification network training process, the training batch size was equal to 84, each class had 28 representatives in the training batch. In addition, at the beginning of each training epoch, representatives for each class were randomly selected from the training set and assigned to the training batch. An equal number of class representatives in the training batch allowed to gain balance in the training process. A the same time, modification of dataset composition during each training epoch allowed to avoid overfitting to the training data. Noteworthy, validation as well as test dataset were defined at the beginning of the training process and were not modified.

In order to augment the dataset, rotation, as well as translation, were applied to each image. The augmentation process is time-consuming therefore to speed up the training phase, a cache with augmented oocytes was created. Each oocyte in the database was rotated 12 times, for *i*th rotation angle was determined according to the following equation 360/12*(i-1). In addition for each rotation, the oocyte was randomly shifted horizontally and vertically in the range of 40px (this type of augmentation was applied 5 times to each rotated item). As a result, the dimension of the input data tensor was expanded to 590 x 590px. Oocyte classification DNN input has a dimension of 590x590xN where N is the number of input classes selected for a given scenario.

#### Segmentation network

The input of the segmentation DNN accepted 512x512px RGB images. Rectangular boxes that enclosed oocytes were extracted from original microscopic images in a way that every part of the oocyte was present in the resulting image. Further images were resized to 512x512 px representation with bilinear filtering. For each stratified repeated random subsampling, the dataset was divided randomly into three classes: training (657 images, 30 MI, 600 MII, 30 PI), validation (15 images, 5 MI, 5 MII, 5 PI), test (91 images, 9 MI, 58 MII, 24 PI). For the dataset augmentation, efficient Pytorch libraries were utilized. Therefore, contrary to the classification approach cache was not created [25, 26].

#### Stratified repeated random subsampling validation

A stratified repeated random subsampling (SRRS) validation procedure was applied in order to evaluate the created models. By default, the data set was divided 3 times. In order to confirm the quality of the final classification structure, the dataset was divided randomly 10 times.

According to the best practices, the dataset is divided into three independent sets that are utilized during the training/evaluation phase: a training set, a validation set and a test set. The training set is utilized in order to fit the model, the validation set is utilized to evaluate the error on the samples that were not used in the training process in order to select potentially the best snapshot of the DNN internal state, and the test set is used at the end of the training phase in order to evaluate the quality of the selected ANN snapshot.

The dataset was imbalanced, it contained a different number of representatives for MI, MII and PI classes, therefore stratification procedure was applied. The purpose was to create validation and test sets with a balanced number of each class representatives.

### Quality coefficients

Below a list of coefficients utilized for the purpose of system quality assessment was provided [27]:

- Mean accuracy coefficient ($\overline{Acc}$) was applied for the purpose of classification DNN structure quality evaluation. It is defined according to the following equation:
  2 $$\begin{aligned} \overline{ACC} = \frac{\sum _{k=1}^{SRRS\_N}{\overline{ACC^{k}}}}{SRRS\_N}, \end{aligned}$$
  where $SRRS\_N$ number of SRRS dataset divisions;
  3 $$\begin{aligned} \overline{ACC^{srrs\_id}} = \frac{\sum _{c=1}^{CLASS\_N}{{ACC_{c}^{srrs\_id}}}}{CLASS\_N}, \end{aligned}$$
  where $CLASS\_N$ - number of oocyte types;
  4 $$\begin{aligned} ACC_{class}^{srrs\_id} = \frac{Tp_{class}^{srrs\_id}}{Tp_{class}^{srrs\_id} + Tn_{class}^{srrs\_id}}, \end{aligned}$$
  where *class* represents oocyte type (MI, MII, PI), $Tp_{class}$ is class true positive, $Tn_{class}$ is class true negative, $srrs\_id$ instance of SRRS dataset division.
- Mean true positive rate ($\overline{TPR}$) describes the relation between true positives and all positive elements across SSRS. It is defined according to the following equation:
  5 $$\begin{aligned} \overline{TPR} = \frac{\sum _{k=1}^{SRRS\_N}{\overline{TPR^{k}}}}{SRRS\_N}, \end{aligned}$$
  where $SRRS\_N$ number of SRRS dataset divisions;
  6 $$\begin{aligned} \overline{TPR^{srrs\_id}} = \frac{\sum _{c=1}^{CLASS\_N}{{TPR_{c}^{srrs\_id}}}}{CLASS\_N}, \end{aligned}$$
  where $CLASS\_N$ - number of oocyte types;
  7 $$\begin{aligned} TPR_{class}^{srrs\_id} = \frac{Tp_{class}^{srrs\_id}}{Tp_{class}^{srrs\_id} + Fn_{class}^{srrs\_id}}, \end{aligned}$$
  where *class* represents oocyte type (MI, MII, PI), $Tp_{class}$ is a class true positive, $Fn_{class}$ is a class false negative, $srrs\_id$ instance of SRRS dataset division.
- Mean positive predictive value ($\overline{PPV}$) describes the relation between true positives and all elements classified as positive, across SSRS. It is defined according to the following equation:
  8 $$\begin{aligned} \overline{PPV} = \frac{\sum _{k=1}^{SRRS\_N}{\overline{PPV^{k}}}}{SRRS\_N}, \end{aligned}$$
  where $SRRS\_N$ number of SRRS dataset divisions;
  9 $$\begin{aligned} \overline{PPV^{srrs\_id}} = \frac{\sum _{c=1}^{CLASS\_N}{{PPV_{c}^{srrs\_id}}}}{CLASS\_N}, \end{aligned}$$
  where $CLASS\_N$ - number of oocyte types;
  10 $$\begin{aligned} PPV_{class}^{srrs\_id} = \frac{Tp_{class}^{srrs\_id}}{Tp_{class}^{srrs\_id} + Fp_{class}^{srrs\_id}}, \end{aligned}$$
  where *class* represents oocyte type (MI, MII, PI), $Tp_{class}$ is class true positive, $Fp_{class}$ is class false positive, $srrs\_id$ instance of SRRS dataset division.
- Mean false discovery rate ($\overline{FDR}$) which is the expected proportion of type I error, across SSRS. It is defined according to the following equation:
  11 $$\begin{aligned} \overline{FDR} = \frac{\sum _{k=1}^{SRRS\_N}{\overline{FDR^{k}}}}{SRRS\_N}, \end{aligned}$$
  where $SRRS\_N$ number of SRRS dataset divisions;
  12 $$\begin{aligned} \overline{FDR^{srrs\_id}} = \frac{\sum _{c=1}^{CLASS\_N}{{FDR_{c}^{srrs\_id}}}}{CLASS\_N}, \end{aligned}$$
  where $CLASS\_N$ - number of oocyte types;
  13 $$\begin{aligned} FDR_{class}^{srrs\_id} = \frac{Fp_{class}^{srrs\_id}}{Fp_{class}^{srrs\_id} + Tp_{class}^{srrs\_id}}, \end{aligned}$$
  where *class* represents oocyte type (MI, MII, PI), $Fp_{class}$ is class false positive, $Tp_{class}$ is class true positive, $srrs\_id$ instance of SRRS dataset division.
- Mean intersection over union ($\overline{IoU}$) was used for the purpose of the segmentation network quality assessment. The coefficient for $\overline{IoU}$ was calculated according to the following equation:
  14 $$\begin{aligned} \overline{IoU} = \frac{\sum _{k=1}^{SRRS\_N}{\overline{IoU^{k}}}}{SRRS\_N}, \end{aligned}$$
  where $SRRS\_N$ number of SRRS dataset divisions;
  15 $$\begin{aligned} \overline{IoU^{srrs\_id}} = \frac{\sum _{c=1}^{CLASS\_N}{{IoU_{c}^{srrs\_id}}}}{CLASS\_N}, \end{aligned}$$
  where $CLASS\_N$ - number of oocyte types;
  16 $$\begin{aligned} IoU_{class}^{srrs\_id} = \frac{Tp_{class}^{srrs\_id}}{Tp_{class}^{srrs\_id} + Fp_{class}^{srrs\_id} + Fn_{class}^{srrs\_id}}, \end{aligned}$$
  where *class* represents oocyte type (MI, MII, PI), $Tp_{class}$ is class true positive, $Tn_{class}$ is class true negative, $srrs\_id$ instance of SRRS dataset division.
- Detection ratio ($DR$) is the ratio between regions of the oocyte that were correctly detected (at least 100 px of the region was detected correctly) to the number of oocytes in the validation or test set.

#### FLOPs determination

Floating point operations (FLOPs) are used commonly for the purpose of computational complexity neural network assessment. FLOP term refers to a single arithmetic operation performed on floating-point numbers, among these operations are: additions, subtraction, multiplication and divisions.

In order to calculate FLOPs, fvcore tool was utilized. Tool was implemented in Python, therefore DNNs generated in Matlab were converted to onnx format then imported to Pytorch and analyzed by fvcore.

## Data Availability

Trained models as well as code which allow running the processing pipe are available under https://github.com/dmyszor/Embriology.

## Abbreviations

Acc: Accuracy
$\overline{Acc}$: Mean accuracy coefficient
AI: Artificial intelligence
ANN: Artificial neural network
ART: Assisted reproductive technology
CNN: Convolutional neural network
COC: Cumulus–oocyte complex
DNN: Deep neural networks
$\overline{FDR}$: Mean false discovery rate
FFPB: Fragmented first polar body
FLOPs: Floating operations
FPB: First polar body
GV: Germinal vesicle
ICSI: Intracytoplasmic sperm injection
IVF: In vitro fertilization
IoU: Intersection over union
$\overline{IoU}$: Mean intersection over union
MII: Metaphase II stage oocytes
MI: Metaphase I stage oocytes
NEBD: Nuclear envelope breakdown
PI: Prophase I stage oocytes
$\overline{PPV}$: Mean positive predictive value
PVS: Perivitelline space
SRRS: Stratified repeated random subsampling
$\overline{TPR}$: Mean true positive rate
ZP: Zona pellucida
